# Wireless Prototype Based on Pressure and Bending Sensors for Measuring Gate Quality

**DOI:** 10.3390/s130809679

**Published:** 2013-07-29

**Authors:** Florent Grenez, María Viqueira Villarejo, Begoña García Zapirain, Amaia Méndez Zorrilla

**Affiliations:** 1 Computer Science Department, ICAM University, 6 rue Auber-59000 Lille, France; E-Mail: florent.grenez@orange.fr; 2 DeustoTech-Life Unit, Deusto Institute of Technology, University of Deusto, Avda. de las Universidades,24, Bilbao 48007, Spain; E-Mail: mviqueira@deusto.es (M.V.V.) mbgarciazapi@deusto.es (B.G.Z.)

**Keywords:** gait analysis, pressure sensors, bending sensors, Arduino, multiple sclerosis

## Abstract

This paper presents a technological solution based on sensors controlled remotely in order to monitor, track and evaluate the gait quality in people with or without associated pathology. Special hardware simulating a shoe was developed, which consists of three pressure sensors, two bending sensors, an Arduino mini and a Bluetooth module. The obtained signals are digitally processed, calculating the standard deviation and establishing thresholds obtained empirically. A group of users was chosen with the aim of executing two modalities: natural walking and dragging the left foot. The gait was parameterized with the following variables: as far as pressure sensors are concerned, one pressure sensor under the first metatarsal (right sensor), another one under the fifth metatarsal (left) and a third one under the heel were placed. With respect to bending sensors, one bending sensor was placed for the ankle movement and another one for the foot sole. The obtained results show a rate accuracy oscillating between 85% (right sensor) and 100% (heel and bending sensors). Therefore, the developed prototype is able to differentiate between healthy gait and pathological gait, and it will be used as the base of a more complex and integral technological solution, which is being developed currently.

## 1. Introduction

The analysis of the gait [1] has different applications: it can be used for athletes to improve their capacity [2] or it can be applied in terms of health [3].

This study aims to develop a technological solution to evaluate the walking performances of patients with neurological diseases that affect their gait. Some of these diseases could be Parkinson, multiple sclerosis (MS) or palsy.

For the diagnosis and evaluation of multiple sclerosis, specialists use a method known as Multiple Sclerosis Functional Composite (MSFC) [4]. One of the tests consists of asking the patient to walk a distance of 25 feet (Timed 25-Foot Walk) at the specialist consultation, while the doctor calculates the time the patient needs to complete the test. Subsequently, the practitioner asks different subjective questions to the patient about their physical state.

The aforementioned test presents two disadvantages. On the one hand, it does not obtain objective data regarding the quality of the footstep. On the other hand, it is too short, so it does not reflect the patient's answer to daily route; thus, it is not possible to make a comparison of the gait quality during the day.

The main objective of this study is to develop a portable device that acquires different signals and parameters for their subsequent monitoring with the aim of evaluating gait quality.

The device would be able to record all the data for a gait analysis all day, while the patient is doing his everyday activities without noticing it.

This first prototype is a shoe with integrated sensors that measure different gait patterns: pressure sensors and bending sensors. The final objective is to develop a device that can help doctors evaluate the evolution of the disease or the treatment effectiveness through the evolution of the way of walking. Additionally, the final device would help doctors to obtain objective results, allowing for comparison of a patient, with another aiming for the construction of a database. Furthermore, it will allow doctors to obtain data from the patients while they performing their everyday life activities.

The first test protocol was defined following the test that professionals who study sclerosis perform with their patients. This test consists of asking the patient to walk straight for 7.5 meters and then come back. The patient is allowed to walk with all the devices normally used, but it is not possible to be aided with a wall or the doctor's arm.

The aim is to carry out exactly the same test, the only difference being the fact that at the moment, only the time that the patient takes to achieve this test is evaluated in terms of precision. The gait analysis is made with the eye and taking into account the doctor's experience. Thus, it can be said that the test is really subjective. The final device will study the foot pressure, the articulation angles and the foot motion, so it will result in more objectivity.

This project uses the Arduino technology for the microcontroller and Bluetooth protocol as the wireless technology. Different sensors were chosen to achieve this objective: pressure sensors and angle sensors.

The hardware was initially tested technically for the subsequent integration in a prototype in order to procure medical evidence. These tests were carried out by healthy people walking normally and dragging their feet.

The results of these tests led to the creation of a series of indicators the doctors could use. The next step for the prototype is to be integrated in a real shoe with more sensors, such as an accelerometer, gyroscope, thermometer, *etc.* The final prototype will be tested by real patients in order to verify that the indicators chosen are usable.

The contents of this document can be summarized as follows: First of all, the state-of-the-art with the different studies and technologies will be presented. Secondly, the methods with the used Hardware and communications, followed by the system design explaining the different parts will be presented. Then, the obtained results and, finally, the discussion, conclusion and the acknowledgments will be presented.

### 1.1. State-of-the-Art

Nowadays, more research labs, hospitals and companies are getting interested in baropodometry, which is the analysis and evaluation of the foot position and the weight distribution on the foot during walking. A study of the human gait is described in [5] and a state-of-the-art wearable sensor is shown in [6].

In [7], Ebersbach *et al.* compare the gait between Parkinson, ataxia and subcortical arteriosclerotic encephalopathy. The subjects were asked to walk with different speeds. The results show that patients with subcortical arteriosclerotic encephalopathy presented more differences with respect to the healthy controls. There are numerous studies of the gait in patients with MS. In [8], the authors use Pearson correlation and cross tabulation to determine difference between MS and the control patients, extracting the different data from a mat with integrated sensors. Sehle *et al.* [9] present a study of the gait patterns aiming at the objective assessment of fatigue. Gehlsen *et al.* [10] study whether changes of gait quality can be appreciated in people with sclerosis while they are engaging in an aquatic experiment.

As far as the hardware part is concerned, there are numerous prototypes and studies that integrate the sensor in a shoe or by using an insole.

The baropodometric insole is equipped with sensors in the same way as a baropodometric walking mat. That way, the patient is able to walk wherever he wants and can even wear the insole in different situations. It is much easier to get the patient to walk for a long time, the way of walking not being disrupting. The study in [11] was made with patients with diabetes using the system F-scan, which detects the pressure made by the users wearing their own shoes. The authors describe in [12] a textile with integrated sensors that sends the data to a subsequent application to analyze them.

The major problem concerning these insoles is that they cannot be used without a shoe or any other system to maintain them under the foot. In fact, there is always another real sole under this insole, which is helpful for determining the shoe absorption and to compare the results pf walking barefoot obtained by other devices. The insoles are widely used to help in designing shoes for a special activity, due to the fact that by means of the sole, it is possible to determine exactly which part of the shoe is undergoing the most pressure. Then, it is possible to adapt the shoe to this particular activity; this could be made for different reasons, such as to reinforce it.

Baropodometric soles also present the inconvenience of being very fragile, and they stop working rapidly. Apparently, according to Fany Chedevergne in her thesis on baropodometric devices [13], it is advised not to make use of them more than six to 20 times, depending on the technology used. For their appropriate utilization, it is necessary to cut the sole at the right patient size, which makes it useless for the next one.

Shoes with integrated sensors have the advantage of a baropodometric sole; since the shoe is fixed to the foot, the user is able to make as many steps as wanted with the sensors, and it does not affect the patient's gait. On the contrary, they do not present the main disadvantages of the sole. In fact, if the sensors are placed all the way through the sole, the pressure between the floor and the foot is measured directly: the sole is only there to maintain the sensor. This solution seems better than fixing the sensor directly on the foot, since it ensures that the sensors are always at the same place, which is more interesting if it is desired to compare the results with other patients. The inconvenience for the patient occurs when it is wanted to wear them during everyday activities. It will be necessary to wear these shoes, because they cannot fit in everyday shoes, such as insoles. Another problem resides in the fact that the shoe is made only for a special size, which restricts the number of users. Nevertheless, it should be possible to make removable sensors, which means it could be possible to make different sizes of shoes with the sensors emplaced, using only one set of sensors placed in the selected shoes depending on the patient foot size. In [13], a list of shoes made with integrated sensors can be seen.

In 2004, Morris and Paradiso made shoes that were even more complete ([14,15]). These shoes contained two gyroscopes axes and two accelerometers axes on each shoe in order to evaluate the foot motion. It also contained four force sensitive resistors on each shoe: two of them measured the heel pressure (right and left), another one measured the pressure on the first metatarsal and the last one measured the fifth metatarsal pressure. There were four bending sensors working in pairs to measure the ankle angle and the angle between the metatarsals and the phalanges. Two bending sensors were needed for one angle, since they only measure a positive angle, in opposite directions. Morris and Paradiso also used two polyvinylidene fluoride stripes on each foot (one under the heel and the other one under the hallux) for evaluation of the force exerted on the heel when it hits the floor and the force exerted on the hallux when it leaves the floor. Eventually, they used an electric field sensor on each foot to measure the distance between the foot and the floor, as well as an ultrasound sensor to get the distance between the two feet during walking. The ultrasound sensors contained one transmitter and two receivers; thus, the angles and distances between them were used to calculate the distance between the two feet.

After that, Faivre designed shoes to measure the plantar pressure on the floor [16]. This means that the sensors were only between the foot and the floor, because the author thought that most of the insole gait sensors measured the plantar pressure inside the shoe and not the bare foot. To achieve that, a special sensor based on a deformable ring was designed. In such a way that when the patient walks, the ring is pressed and deformed, so Faivre placed sensors on this ring (deformation gauges) to measure the deformation. This way, the foot pressure was able to be calculated. This shoe was then further developed by Chedevergne in order to make it usable for a wider range of patients by changing the ring properties, as well as removing the sensor casing and adding more sensors. She also lightened the backpack hosting the data treatment device.

## 2. Methods

### 2.1. Hardware

As stated before, it is important for practitioners to know the gait evolution during the day. For this reason, it was opted to develop a system based on a shoe, using Arduino and different sensors.

#### 2.1.1. Arduino

**Figure 1.**
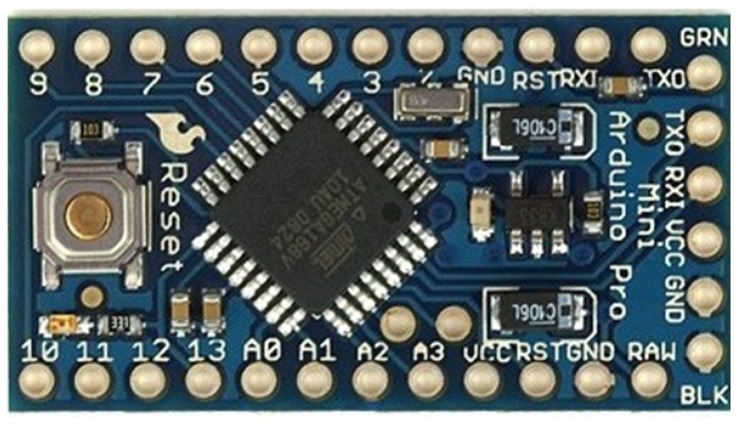
Arduino Mini.

Arduino Mini of 3.3 V (Figure 1) was chosen, due to its dimensions and its facility to be integrated in the shoe.

The Analogue Converter of this Arduino is 10 bits, so it is possible to measure data from 1 to 1,024 in a range between 0 and 3.3 V. The resolution is the following:

$$\text{Resolution}=\frac{3.3}{1024}=3.22mV$$

#### 2.1.2. Sensors

Two kinds of sensors were used for this initial test: pressure sensors and bending sensors. A review of foot plantar pressure measurement systems is described in [17].

##### Pressure Sensors

This kind of sensors was used to obtain the plantar pressure under the feet. It was opted to use a piezoresistive pressure sensor. These sensors are made of three deformation gauges in three different and orthogonal directions put into a silicone gel. Under pressure, the gel is deformed, and the gauges measure this deformation. Knowing the deformation gauge and the gel characteristics, it is possible to calculate the pressure from the deformation. These sensors are preferred, because of their excellent linearity and their good reactivity.

**Figure 2.**
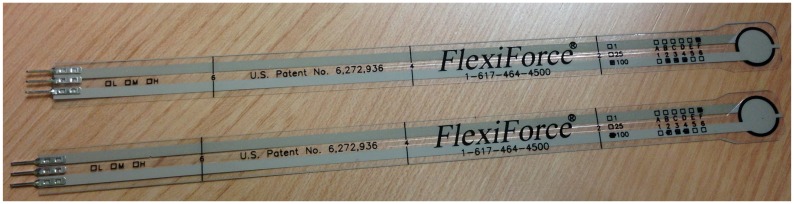
Flexiforce pressure sensor, 100 lbs.

The prototype integrates Flexiforce pressure sensors of 100 lbs (see Figure 2). These sensors were used in previous studies [18]. Zhang *et al.* [19] developed a plantar system to know the pressure made by the user, and they also including an explanation of the technical features.

The pressure sensors are thin and very flexible resistive sensors. They work like variable resistors: the resistance depends on the thickness of the sensor. Thus, when there is a force applied to the sensor, the thickness gets lower, so that the resistance drops down, as well.

**Figure 3.**
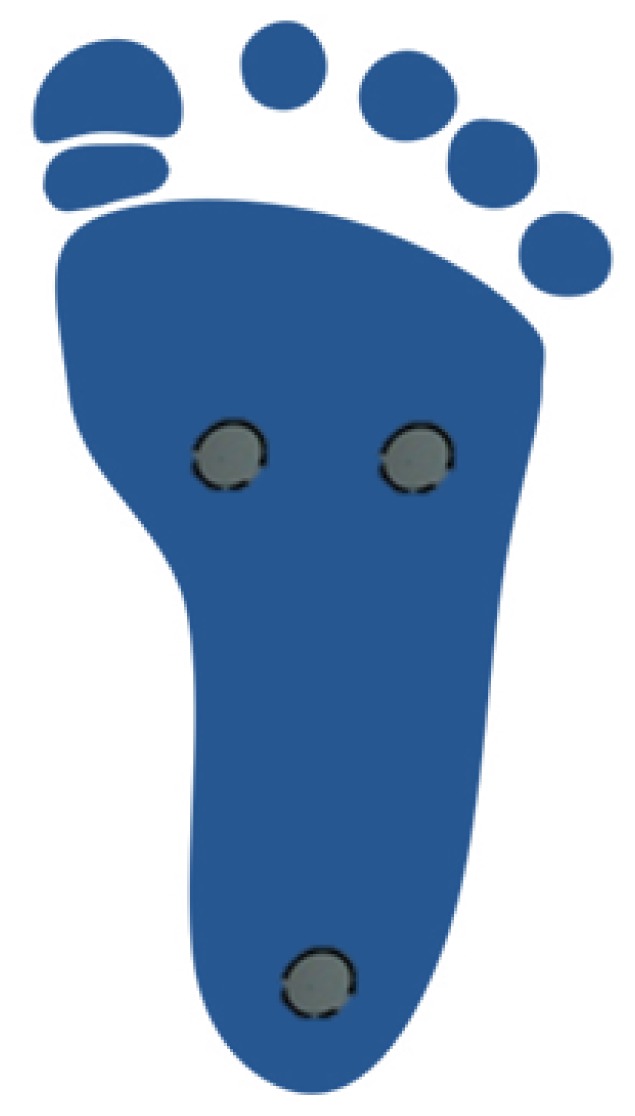
Placement of the pressure sensors.

Three pressure sensors were disposed: one under the first metatarsal, another one under the fifth metatarsal and the last one under the heel (Figure 3)

##### Bending Sensors

**Figure 4.**
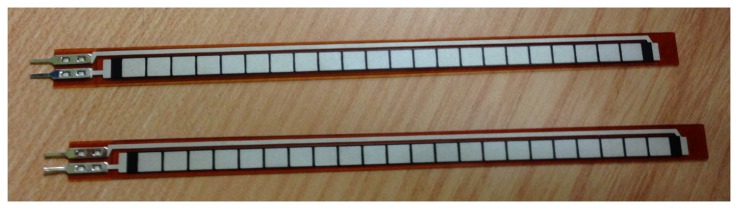
Flex sensor, 4.5″.

**Figure 5.**
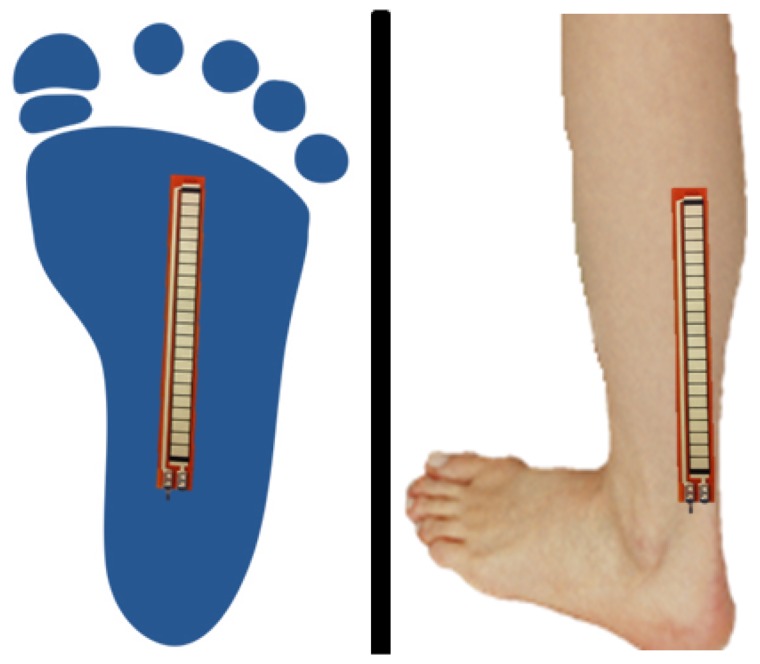
Placement of the angle sensors.

In order to study the foot motion, it is very interesting to know the angle formed by the ankle and the metatarsals. For that reason, it was opted to add two bending sensors shown in Figure 4: one of them under the sole of the foot and the other one around the ankle; Figure 5.

Bending sensors see that their electrical resistance changes when they are bent. They are flexible sensors that are resistive, as well. In fact, when they are bent, the material composing them is stretched, so the current going through it has a longer way to go. When the sensor is bent, the sensor resistance rises, depending on the angle. In [20], Kappel *et al.* present a strain sensor, based on PolyPower, which measures the strain between two points.

**Figure 6.**
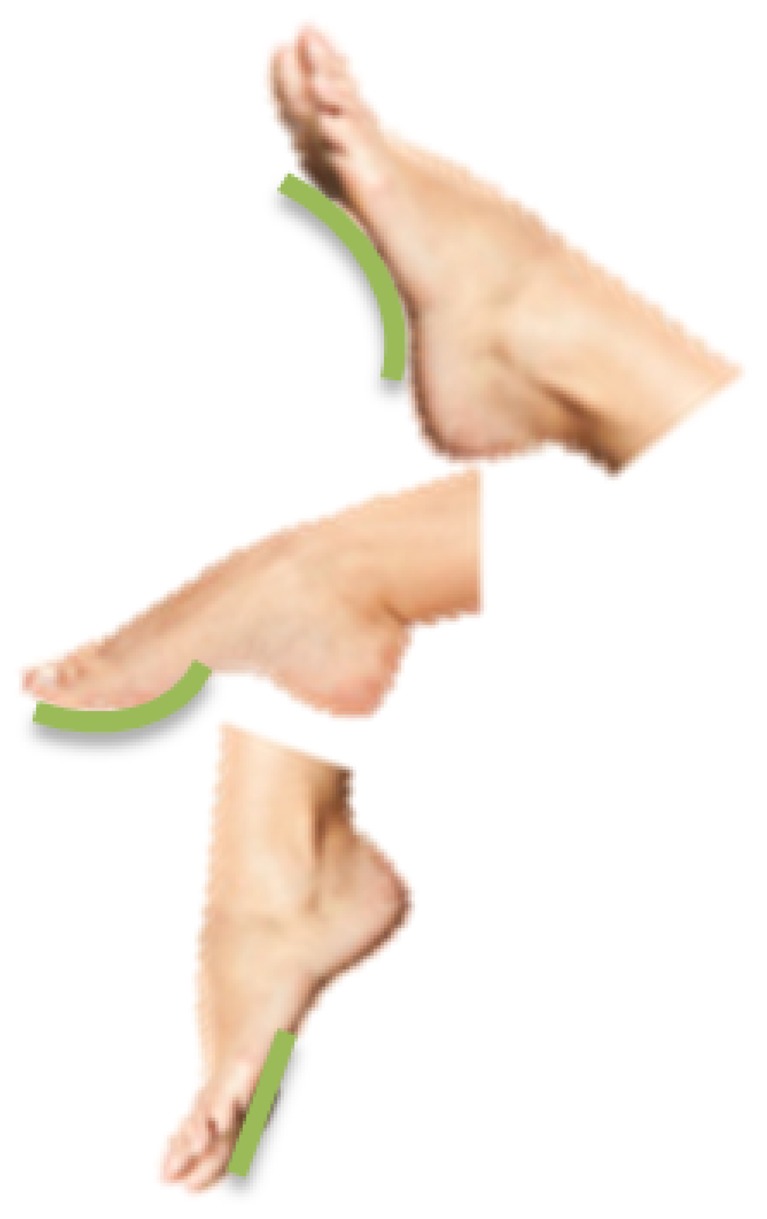
Foot movements.

The bending sensor placed in the shoe sole (see Figure 6) detects the plantar and the dorsal flexion when the user walks. The first of the movements increases the angle between the frontal part of the foot and the tibia, whereas the dorsal flexion is the inverse movement: it decreases the angle, the foot approaching the tibia.

#### 2.1.3. Circuits

**Figure 7.**
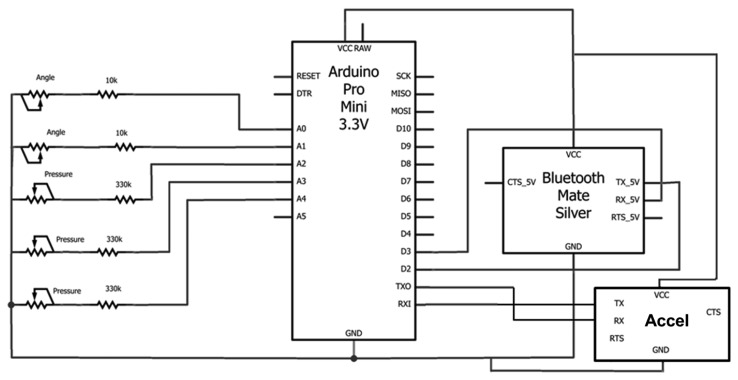
General circuit.

This section shows the different parts of the circuit built for this first prototype. The general circuit is shown in Figure 7.

Arduino evaluates the voltage; thus it is necessary to build a voltage divider with a chosen resistance. That way, it is possible to evaluate the sensor resistance and, by this, deduce the force applied to the sensor.

##### Pressure Sensors

**Figure 8.**
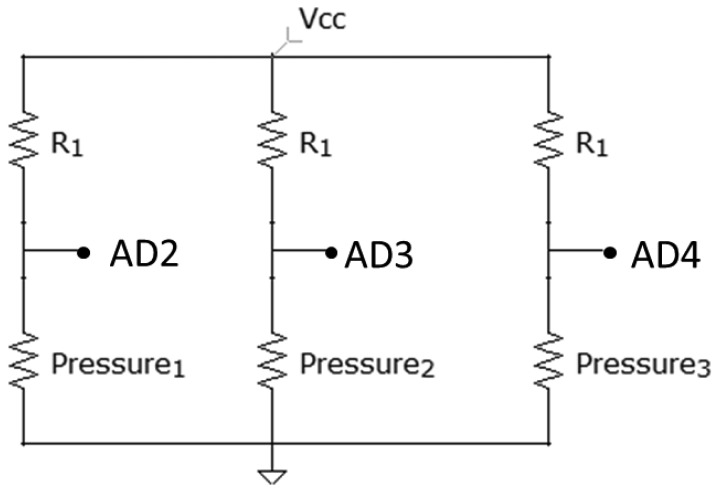
Pressure sensors circuit.

In Figure 8, it can be seen how the three pressure sensors are connected. The value of the resistances, R1, is 330 kΩ. AD2, AD3 and AD4 correspond to three of the analogical inputs of Arduino.

The output value of each line is:

$$Vo=\frac{\text{Pressurex}}{\left(R1+\text{Pressurex}\right)}\text{Vcc}$$

It can be concluded that, the more pressure, the more output voltage there will be. However, the different obtained voltages were very similar, undertaking additional operations in order to obtain higher differences between each value:

$$\text{Value}=\frac{100}{3Vo}$$

##### Bending Sensors

**Figure 9.**
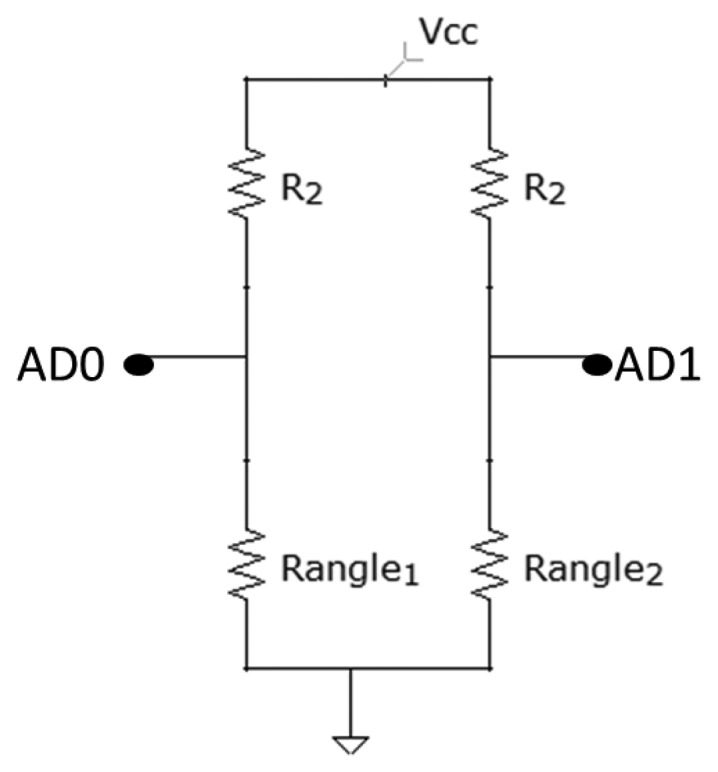
Bending sensors circuit.

Figure 9 shows the circuit for the bending sensor connection. The value of the resistances, R2, is 10 kΩ.

The bending sensor has a resistance, which varies from 10 to 40 K, making it necessary to have a tension dividing bridge with a resistance around the same value to measure the resistance changes caused by the bending.

$$Vo=\frac{\text{Ranlge}}{\left(R2+\text{Rangle}\right)}\text{Vcc}$$

$$\text{Rangle}=R2\frac{\frac{Vo}{\text{Vcc}}}{1-\frac{Vo}{\text{Vcc}}}$$

**Figure 10.**
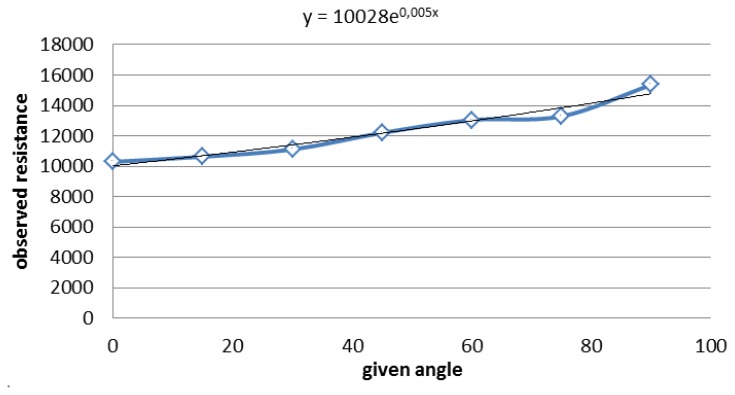
Resistance depending on the angle.

In order to find a link between the sensor's angle and the resistance, it is necessary to give a particular known angle and compare it with the output of the sensor. A lot of measures for the same angle were made, and the average was taken. Then, through all this data, it is possible to draw the Figure 10.

Furthermore, it is known, from the sensor data sheet given by the supplier, that the curve should be an exponential one. Hence, using excel, a tendency curve that gives the equation was drawn, as can be seen on the graph.

From the equation, the angle depending on the resistance can be deduced. The angle of the sensor, deduced from Figure 10, presents the following equation:

$$\text{angle}=\frac{ln(\text{Rangle})-ln(10028)}{0.005}$$

### 2.2. Communications

The Arduino mini acquires the different signals from the sensors. After that, it sends a string with the different data via Bluetooth to the computer in order to be shown and processed.

**Figure 11.**
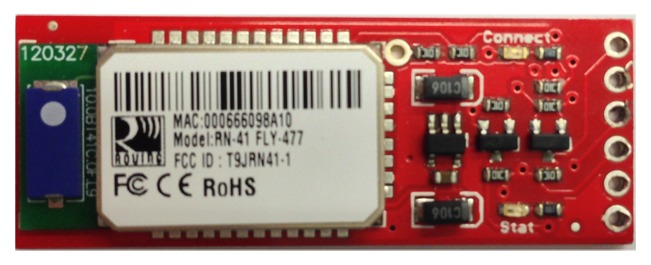
Bluetooth Mate Gold.

The Bluetooth module is called Mate Gold (Figure 11). It was chosen because of its comfortability at the time of connecting to the Arduino.

The data were collected by Visual Studio using C, while the interface and the processing stage were developed with Matlab. The schemes of the Arduino and the PC can be seen in *Proposed System*.

**Figure 12.**
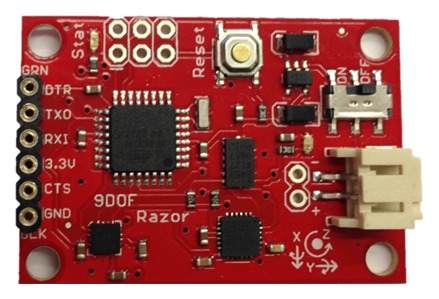
IMU razor.

Although, in this first study, only the pressure and the angles are measured, this first prototype includes one accelerometer for their posterior treatment. The chosen device is the Inertial Measurement Unit (IMU) Razor 9 DOF (see Figure 12). It contains three ac-celerometers, three gyroscopes and three magnetometers. The literature shows different gait studies that integrate accelerometers and gyroscopes [21–23]. They can also be used as pedometers [24]; thus, existing studies compare these two devices [25]. A review of the accelerometers on wearable systems is showed in [26].

**Figure 13.**
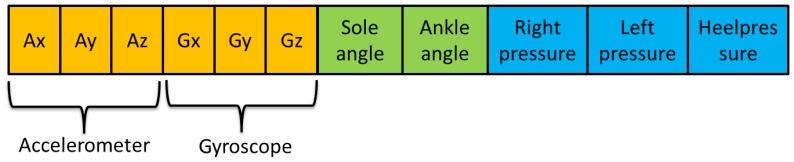
Frame sent by Arduino.

This accelerometer was chosen due to the fact that the parameters are able to be read and its ease of being programmed. The IMU station works independently, and it only sends its results to the Arduino via the serial port. After that, the Arduino links the data from the analogical inputs and the accelerometer; Figure 13.

The accelerometer conditions the sample rate of the prototype, since it is necessary to ensure that the accelerometer sends the all the data before Arduino sends the complete string. After different experimental data, it was verified that the difference between each sample must be 219 ms. That way, the sample rate is, approximately, 4.56 Hz, and the transmission baud rate of the Bluetooth must be 57,600 baudios.

### 2.3. Proposed System

**Figure 14.**
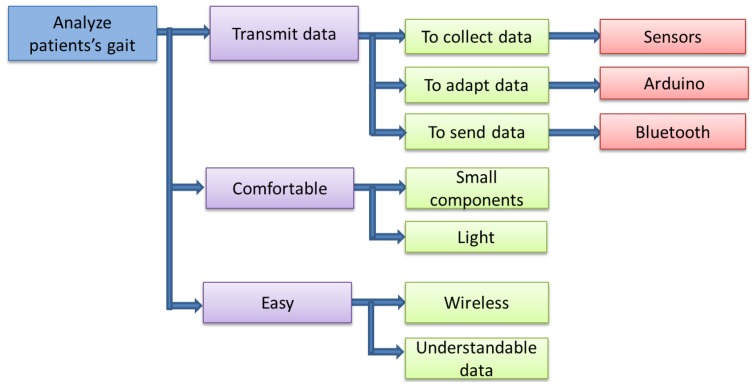
Design specifications.

This section describes the different functions and parts required by the device. It starts with the general functions (upper level), finishing with a more detailed explanation of them. The prototype was designed following the steps shown in Figure 14.

The first of the three main functions is to transmit the data. To achieve this, the device has to fulfill three smaller functions, which are: to collect, analyze and adapt the data and to send them. The second main function is not to disturb the patient's gait, which means that it must be light and have the smallest components as possible. The third function is to be easy in terms of use. For this reason, the device must be wireless, in such a way that the patient can use it anywhere. Moreover, the device must be easy to use by a doctor. Hence, the final data must be presented in curves or figures that can be easily understandable.

#### 2.3.1. Upper Level

**Figure 15.**
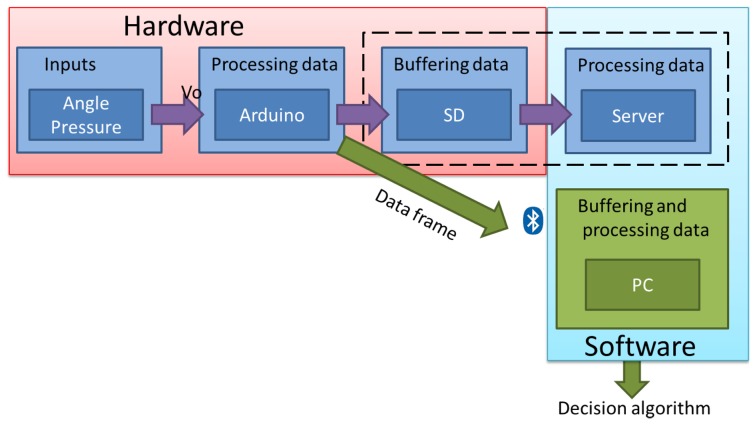
General scheme.

Taking into account the different specifications explained before, the prototype was designed based on the scheme shown in Figure 15.

In this first study, the data are stored on a PC directly in order to obtain a decision algorithm. The final device will store the data on a micro SD for their subsequent treatment in a server. This also means that the final prototype will not include a Bluetooth module, aimed at making the storage of data more reliable.

**Figure 16.**
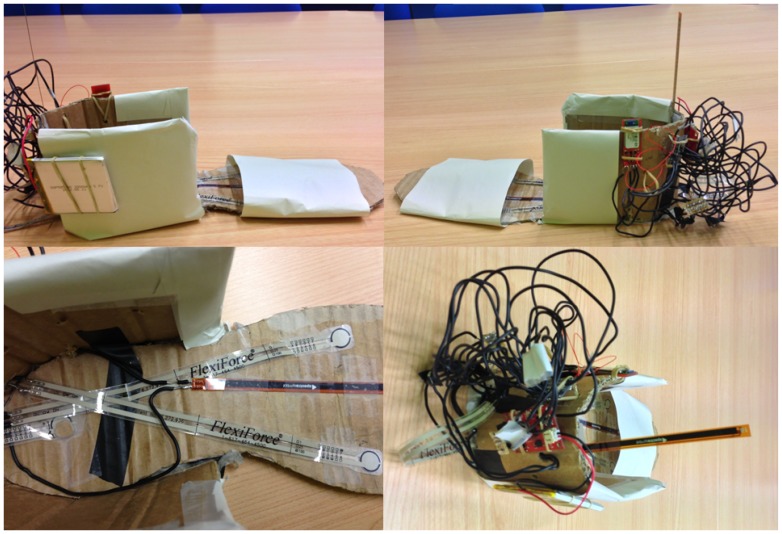
First shoe prototype.

Figure 16 shows the design of the first prototype used for the initial tests.

The placement of the different sensors was changed for each user, taking into account that the pressure sensors are placed under the first and fifth metatarsal and under the heel. The sole sensors are covered for more comfortability for the users.

#### 2.3.2. Low Level

**Figure 17.**
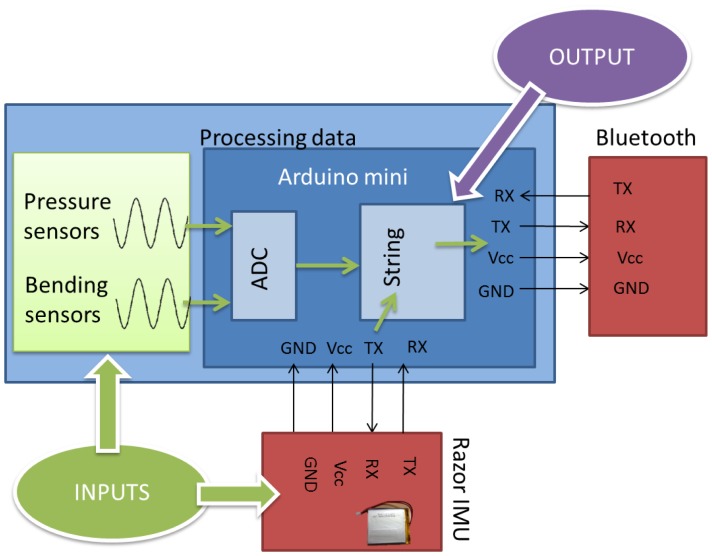
Arduino functions scheme.

**Figure 18.**
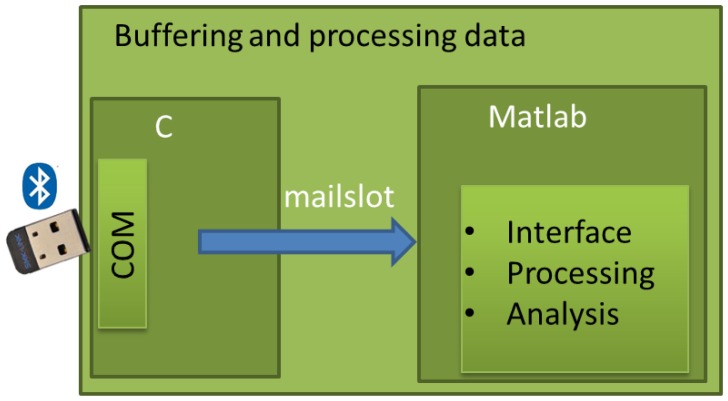
PC scheme.

The schemes of the two blocks with processing stages are shown in Figures 17 and 18. The communication between the Bluetooth and Arduino is done by serial port. Two of the digital pins are used as RX and TX, the receiver transmitter lines for the communication.

The power supply of the device is provided through the accelerometer, using a polymer lithium ion battery. Each time the Arduino receives the data from the accelerometers, it includes the data from the sensors and joins them together in a string.

In the case of the PC, a commercial Bluetooth receiver was used, so Figure 18 does not show any physical connection.

## 3. Experiment Results

**Figure 19.**
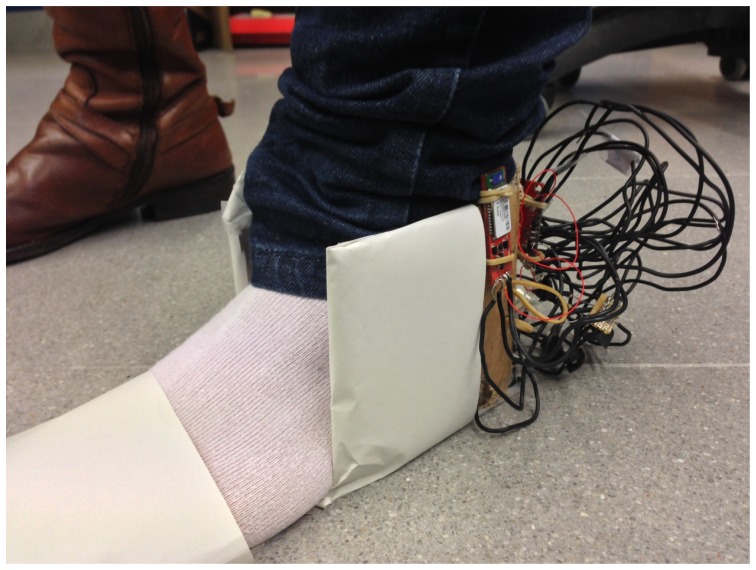
User wearing the prototype.

The shoe was tested with seven users of the work unit, one woman and six men, with ages between 22 and 26. Figure 19 shows one of the users wearing the prototype on the left foot.

**Figure 20.**
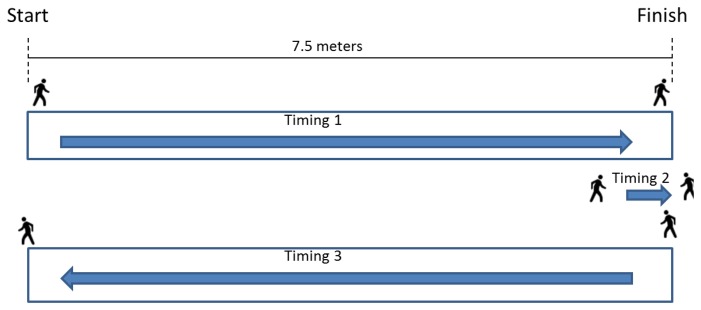
Third and fourth test.

As there was not any person with gait problems, the people who tested the device were asked to do the test walking normally and, then, to do it again, dragging their foot. Finally, the protocol was defined:

First test: The person is standing, but not moving. This enables one to have a basis, especially for the pressure sensors. It gives the pressure value the sensors give when there is no other force applied on them than weight.
Second test: The person is taking six steps, paying special attention to take three full steps with the left foot, this being the the only one being tested. This enables one to check that the sensors are working properly and gives a sample of normal walking.
Third test, Figure 20: The person walks 7.5 m and then back again another 7.5 m. The person is asked to walk normally. At the same time, there is a timing of the three different stages of the test: the first 7.5 m, the time the patient takes to turn around, and the second 7.5 m.
Fourth test: This is exactly the same test as the third one, but the user drags a foot.

### 3.1. Qualitative Analysis

**Figure 21.**
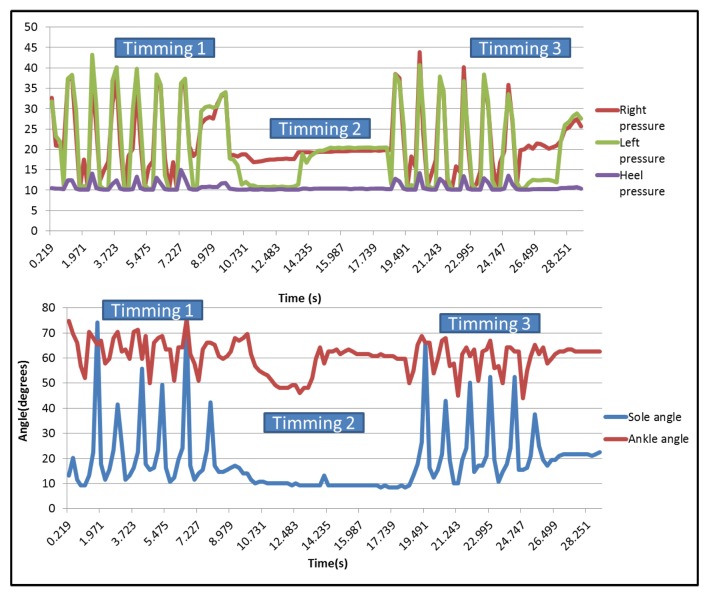
Pressure and bending sensors curve of normal walking.

After collecting the data, it is possible to draw the curves related to each sensor on a graph. For the qualitative analysis, only these graphs are used to evaluate the differences between the dragging walk and the normal walk. Figure 21 shows the pressure sensors and the two angles when the user is walking normally.

It can be seen that there are three different parts on each one, corresponding, respectively, to the first 7.5 m walk (first peaks area), the time taken to turn around (the flat part in the middle) and the return (second peaks area). In the case of right and left pressure sensors, peaks have a similar value, but the heel presents minor ones. The three pressure sensors present synchronized peaks.

**Figure 22.**
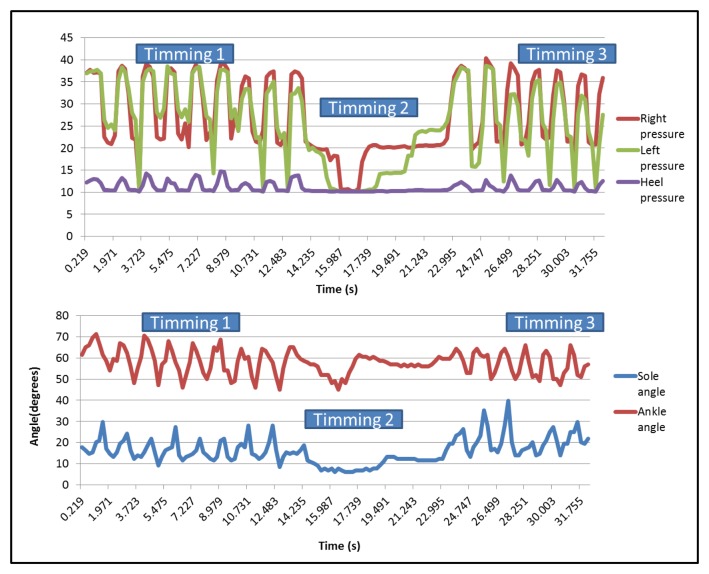
Pressure and bending sensors curves of normal walking.

Figure 22 represents the different pressures and angles when the user is dragging a foot. The graph shows the different steps made by the user during the test.

In this case, the minimum peaks of the right sensor are higher than the graph in Figure 21. During normal walking, the value of them was over 10 (near the heel sensors values), but when dragging a foot, the minimum values were over 20, except for the part in which the user stops to change his position.

#### 3.1.1. Time Analysis

Regarding the time, it is easy to notice that the user takes more time when dragging than when walking normally.

#### 3.1.2. Angle Analysis

**Figure 23.**
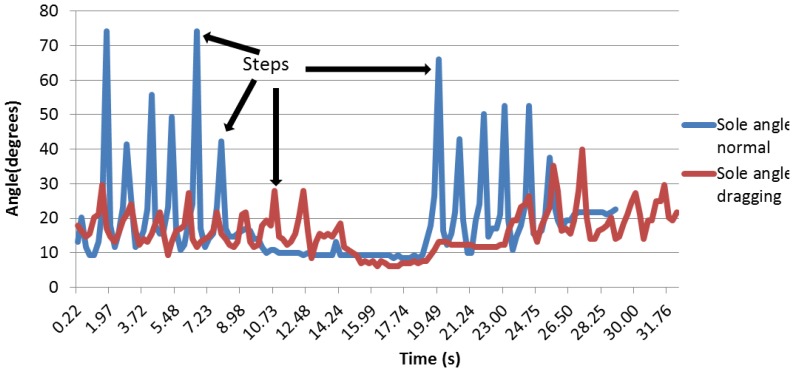
Sole angle curves.

When studying the foot sole angle curve, it can be seen that during a normal walk, the peaks are much higher and much thinner than during the dragging feet walk, at as it can be seen in Figure 23. This can be explained because when dragging, the patient walks slower, so each step takes more time. Thus, the rolling of the foot, which increases the sole angle, takes more time. That is why the peak is wider. The fact that the peak is also smaller during the dragging feet walk can be explained because as the patient is dragging the foot, there is no proper fold, so the angle is smaller.

**Figure 24.**
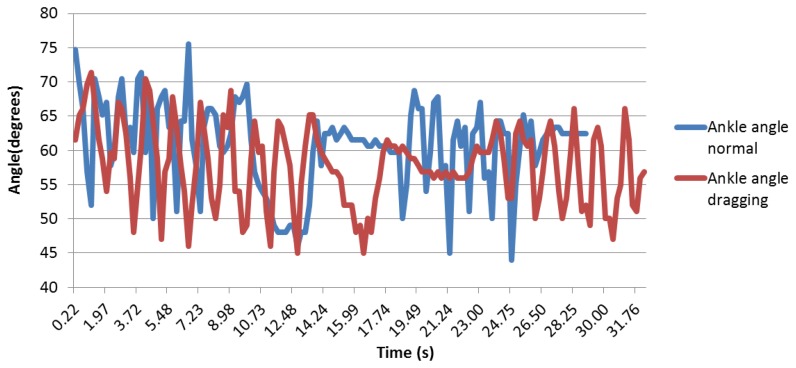
Ankle angle curves.

Regarding the sole angle, it was different for each person, as the foot was not perfectly maintained; thus, it appears that the data are difficult to be compared qualitatively. Figure 24 shows that there is less difference between the normal and dragging peaks.

These curves are better analyzed in the quantitative analysis in order to verify whether there are any differences between them. As the placement of the ankle angle is not fixed in one of its parts, the curves are not as clear as the previous ones.

#### 3.1.3. Foot Pressure Analysis

**Figure 25.**
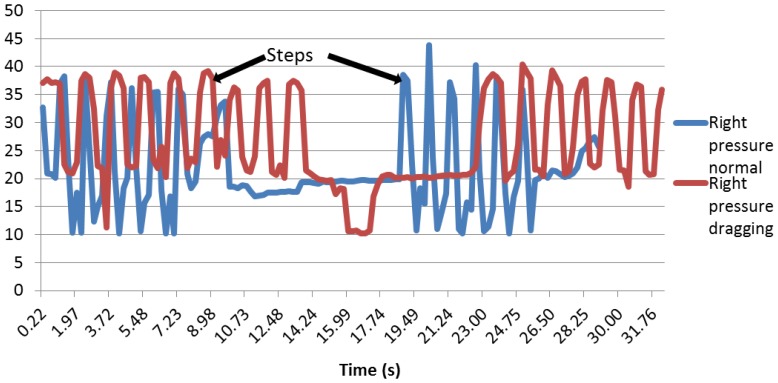
Right pressure sensor.

The foot pressure distribution is interesting, since the graphs show that the patient is well balanced on his foot, since the right and left sensors are detecting a very similar pressure (see Figure 25).

**Figure 26.**
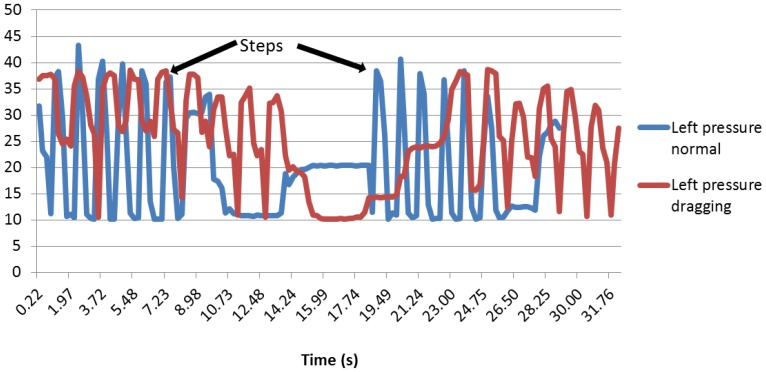
Left pressure sensor.

First of all, as it can be seen in Figure 26, the peaks are starting and stopping at the same time, and the pressure intensity is approximately the same. It is also noteworthy that when the right and left sensors are compared in normal walking and dragging, the curves for dragging are wider than for the normal walk. This can be explained due to the fact that when the patient is dragging the foot, the contact with the floor is longer, so the weight of the body is applied on them for more time.

**Figure 27.**
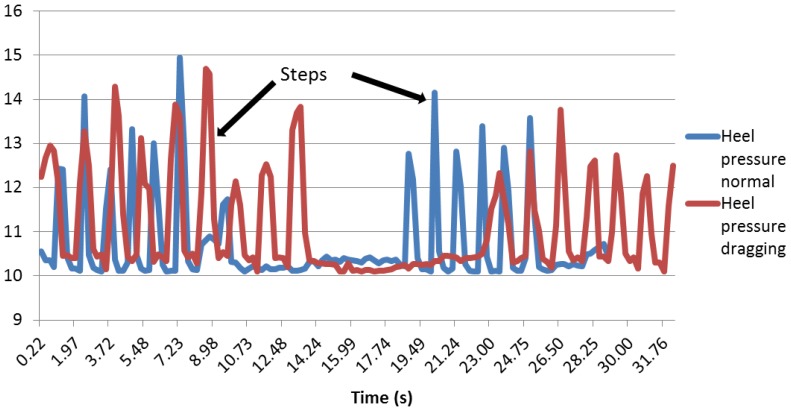
Heel pressure sensor.

As far as the heel pressure sensor is concerned, it can be noticed that the curves are unexpectedly low (see Figure 27). This could be because the heel does not press hard enough on the sensor, so maybe, the sensor has a position that is not the best.

Although the value of the peaks is smaller than the sole and the ankle, the steps are well defined. It can be noticed that the peaks are wider when the user drags a foot, as happened with the other two pressure sensors.

### 3.2. Quantitative Analysis

The qualitative analysis is necessary to get a better understanding of how the walk works, but it is not enough to get certainty. In fact, the qualitative analysis allows a too much subjectivity and interpretation. In this particular field, quantitative analysis is difficult, because each person is different; thus, there is not one precise way to walk, and personal parameters, such as weight, age, speed, health and size, are different for everybody. That is the reason why the confusion matrices are presented.

#### 3.2.1. Time Analysis Indicator

As stated before, the users spent more time on the dragging test, the speed being less than when walking normally. The speed of the users is presented in Table 1.

As there was not any patient with any of the diseases studied available, no particular number can be established, it only can be noticed that when the feet are dragging, the walk is slower. However, this indicator must be mastered by the practitioners.

#### 3.2.2. Angle Analysis Indicator

For this analysis, only the data while the users were walking were taken into account. The stage where the user is turning around to return to the start point is eliminated.

With the aim of evaluating the parameters of the qualitative analysis, it was decided to put into an array the average and standard deviation of the value given by the bending sensor. In fact, it was thought that if the peaks are higher and thinner, then the standard deviation would be higher. The obtained results are shown in Table 2:

The results of the sole angle show that the average of the users is higher when they are walking normally than when they are dragging their foot. It also shows that the dragging feet averages for some users are higher than the normal walking averages for others. Since all the people who have undergone the test are healthy, the average can not be used as an objective quantitative indicator.

For the sole angle, a threshold of 8.5 to distinguish normal walking from dragging was established. All the dragging feet standard deviations are lower than 8.5 and all the normal walking standard deviations are higher than 8.5. In the case of the ankle angle, a threshold of 15 was established. Table 3 shows the confusion matrices:

As far as a healthy person is concerned, if the standard deviation of the sole angle is lower than 8.5, then this person is dragging a foot. This indicator will need to be tested with a future prototype and with diabetic patients or patients with sclerosis or Parkinson to be really reliable.

Table 4 shows the sensitivity, accuracy, specificity and error.

The values of the table, obtained from the confusion matrix, indicate that the thresholds established for the different angle sensors classify the data correctly for all of the users.

#### 3.2.3. Foot Pressure Analysis Indicators

The same phenomenon applies for the sole angle: the peaks are higher and thinner during a normal walk. The same method with the average and the standard deviation was used. The results can be appreciated in Table 5.

Regarding the right pressure sensor, it can be seen that most of the standard deviation values are superior to five when the patient is walking normally and inferior to it when dragging.

In the case of the left pressure sensor, it was noticed that a standard deviation of five would also be a good enough indicator, because most of the dragging foot tests had a standard deviation under five and most of the normal walking tests had a standard deviation higher than five. As can be appreciated, the right and left sensors have the same indicator, since when walking normally and dragging, a patient is always well balanced, aimed at having the body pressure applied equally on both feet.

The heel pressure sensor presents good results. Indeed, as the values were very low, at first, it was thought that they might be wrong, due to the bad position of the sensor. However, in fact, even if the values are low, there is a clear difference between the standard deviation while dragging and a normal walk. The standard deviation for the users dragging their feet is never higher than three, while the standard deviation for the users walking normally is never lower than five. Therefore, it was decided to choose an indicator within that scope, to have a secure safety margin. The chosen indicator is 3.5.

Table 6 contains the confusion matrix for each pressure sensor.

In the qualitative analysis, the right and left sensors had a similar behavior, so the threshold for these sensors is the same. Table 7 shows the different data from the confusion matrix:

In this case, for the established thresholds, Table 7 presents less accuracy than the angle sensors; Table 4; 86%p in the right pressure sensor (the one which presents worst results) and 93% for the left sensor. However, the heel pressure accuracy is 100%, the same as the angle accuracy.

**Figure 28.**
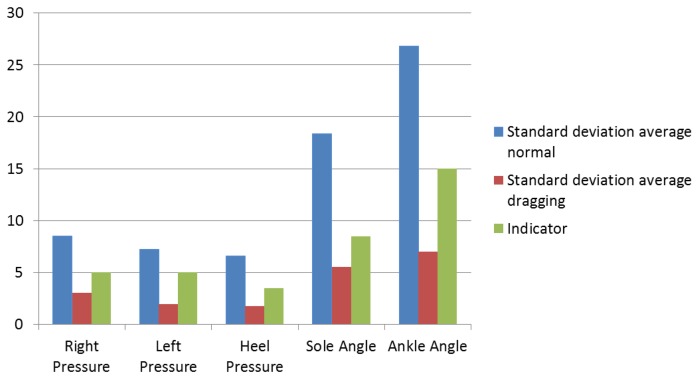
Comparison between the standard deviations average and the indicators.

Figure 28 shows the comparison between the standard deviation average and the indicators. It can be seen that the indicators are always superior to the dragging feet standard deviation and inferior to the average healthy standard deviation. This figure explains the purpose of the indicators, the reference number enabling the doctors to compare their results and help them to see what the patient is suffering from.

The standard deviation average walking normally is much higher for angle sensors than for pressure sensors. In the case of the ankle angle, the qualitative analysis did not show significant differences, but the quantitative analysis shows more differences than the pressure sensors.

## 4. Discussion

A wireless shoe that acquires data from bending and pressure sensors was developed. Thanks to the numerous pieces of literature regarding the state-of-the-art, it was possible to determine which parameters were the best to measure and how to measure them. Seven patients were asked to walk normally and to drag their left foot during a 7.5 m walking test.

This device was made simulating a shoe in which there are three pressure sensors to measure the body weight repartition on the foot and two angle sensors to measure the angle made by the ankle and the sole of the foot. For this project and as a prototype, only one shoe was developed, but to be functional, the device will need two shoes, to be able to compare both feet and to get a full picture of the gait analysis.

The results showed that, by means of the standard deviation, it is possible to establish a threshold to distinguish a person dragging the left foot. This parameter was chosen, because it coincided for all the users: when they walked normally, the standard deviation was higher than when they dragged the foot. It also was observed that the speed of each user is higher when they walk dragging the foot, but it does not establish a common threshold for the different users.

The quantitative analysis reflects a high accuracy of the thresholds for the standard deviation: five for the left and right pressure sensors, 3.5 for the heel, 8.5 for the sole angle and 15 for the ankle angle. The minimum accuracy is 86%, from the right sensor. The left sensor presents an accuracy of 91%, and the rest (heel pressure and bending sensors) present no errors.

There are different studies described in the state-of-the-art that present results at the time of evaluating different parameters of the gait, integrating similar sensors as those used in the present study. In [21], the authors present stride length differences between healthy gait and Parkinsonian gait, presenting differences with regard to the average. In the present study, only the bending sensor of the sole angle presents a difference between dragging and normal walking. The insole developed in [12] was tested standing normally, standing on one leg, heel-strike and push-off, presenting better results than the pressure sensors used in the present study.

The present study does not distinguish the different stages of the gait, but it differentiates between normal gait and dragging a foot with high accuracy. In [27], the authors verify their system based on three pressure sensors and a gyroscope with people with a normal gait and those with a pathological gait. The obtained results show accuracy around 99%, distinguishing the four phases of the gait: stance, heel-off, swing and heel strike. Bae *et al.* [28] presented a system based in ground reaction forces (GRF) to improve the gait quality in patients suffering from Parkinson disease.

This first prototype is a step toward developing a final device that will give objective results to the doctors and that will give the patients the ability to control and supervise their evolution. As the final prototype intends to store the data in a SD target, it will be possible to make a comparison of the gait during the day. It also could be used to improve the gait of different patients.

The hardware will have to work inside a shoe during its use, which means that the sensors and the sensor connections will have to be able to move a little to follow the shoe deformation. Close attention will have to be paid to the electrical connection, because they will have to resist deformations and constraints linked to the shoe environment. The device will also have to be properly integrated in order not to disturb the patient during walking, so that the data are as close as possible to reality.

## 5. Conclusions

This first prototype differentiates between walking normally and dragging the left foot. The five sensors integrated present differences in terms of standard deviation, so it has been possible to establish different thresholds for each sensor. The maximum obtained error corresponds to the right pressure sensor; it is less than the 15%, so the results are good in terms of accuracy.

The future lines of investigation are the following:

- Improve the design of the prototype and test it with real patients;
- Integrate more parameters from different devices;
- Develop the final device, testing it with real patients, storing the data in an SD card and showing them in an application server.

The developed prototype is able to differentiate between healthy gait and imperfect gait and it will be used as the base of a more complex and integral technological solution, which is being developed currently.

## Figures and Tables

**Table 1.** Speed table for the different users.

	**User l**	**User 2**	**User 3**	**User 4**	**User 5**	**User 6**	**User 7**
Timing 1 (s)	8.74	9.73	9.14	9.16	7.95	8.33	9.62
Timing 2 (s)	10.48	11.78	11.4	10.46	10.17	10.32	9.76
Timing 3 (s)	8.81	9	7.59	10.08	7.68	10.19	9.09
**Normal speed (m/s)**	**0.8547**	**0.8547**	**0.8021**	**0.9048**	**0.96**	**0.8182**	**0.8024**
Timing 1 (s)	11.35	10.53	11.04	16.74	12.99	14.83	11.65
Timing 2 (s)	10.35	10.53	10.03	10.6	10.15	10.11	9.82
Timing 3 (s)	10.78	10.94	10.55	17.48	13.37	15.56	12.52
**Dragging speed (m/s)**	**0.6783**	**0.6989**	**0.6951**	**0.4385**	**0.5692**	**0.4939**	**0.6214**

**Table 2.** Average and standard deviation of the angles.

	**Sole angle**						
	**User 1**	**User 2**	**User 3**	**User 4**	**User 5**	**User 6**	**User 7**
**Average-normal**	25.495	26.56	23.497	22.112	22.46	24.977	22.68
**Average-dragging**	24.225	14.744	12.424	18.121	18.709	11.515	16.762
**Sta dev-normal**	19.527	19.281	17.426	17.315	19.243	18.415	17.289
**Sta dev-dragg**	7.972	2.268	3.964	7.624	5.145	3.165	8.759
	**Ankle angle**						
**Average-normal**	53.792	52.899	52.752	30.967	62.238	22.898	48.576
**Average-dragging**	57.08	49.261	54.662	21.151	58.163	48.842	49.049
**Sta dev-normal**	30.711	28.641	26.589	21.221	33.425	21.163	25.778
**Sta dev-dragg**	8.975	10.031	7.549	10.584	6.103	3.07	2.541

**Table 3.** Confusion matrices for bending sensors.

**Sole angle = 8.5**			**Ankle angle =15**		
	**P**	**N**		**P**	**N**
**T**	**7**	**0**	**T**	**7**	**0**
**F**	**0**	**7**	**F**	**0**	**7**

**Table 4.** Statistical data of confusion matrix for the bending sensors.

**Sole angle**		**Ankle angle**	
**Sensitivity**	**1**	**Sensitivity**	**1**
**Accuracy**	**1**	**Accuracy**	**1**
**Specificity**	**1**	**Specificity**	**1**
**Error**	**0**	**Error**	**0**

**Table 5.** Average and standard deviation of the pressure sensors.

	**Right sensor**						
	**User l**	**User 2**	**User 3**	**User 4**	**User 5**	**User 6**	**User 7**
**Average-normal**	11.95	10.795	10.922	16.01	22.323	10.281	14.215
**Average-dragging**	11.936	10.434	11.075	17.85	29.831	10.268	13.674
**Sta dev-normal**	6.678	5.855	5.744	11.087	15.696	5.212	9.28
**Sta dev-dragg**	1.88	0.268	0.622	5.918	7.803	0.075	4.935
	**Left sensor**						
**Average-normal**	10.943	10.86	10.34	10.92	20.855	10.535	11.093
**Average-dragging**	11.533	11.864	10.428	11.182	28.417	10.215	11.003
**Sta dev-normal**	6.008	5.962	5.265	5.837	15.78	5.534	6.142
**Sta dev-dragg**	2.118	1.264	0.307	1.26	7.716	0.118	0.964
	**Heel sensor**						
**Average-normal**	12.351	11.034	10.949	11.441	10.848	11.354	11.566
**Average-dragging**	12.485	11.415	11.19	11.513	11.357	12.135	11.918
**Sta dev-normal**	7.989	6.342	5.998	6.702	6.023	6.512	6.681
**Sta dev-dragg**	2.888	1.191	1.169	1.761	1.158	1.955	2.06

**Table 6.** Confusion matrix for the pressure sensors.

**Right = 5**			**Left = 5**			**Heel = 3.5**		
	**P**	**N**		**P**	**N**		**P**	**N**
**T**	**7**	**0**	**T**	**7**	**1**	**T**	**7**	**0**
**F**	**2**	**5**	**F**	**0**	**6**	**F**	**0**	**7**

**Table 7.** Statistical data of confusion matrix for the pressure sensors.

**Right**		**Left**		**Heel**	
**Sensitivity**	1	**Sensitivity**	O.88	**Sensitivity**	1
**Accuracy**	0.8	**Accuracy**	0.93	**Accuracy**	1
**Specificity**	0.71	**Specificity**	1.00	**Specificity**	1
**Error**	0.14	**Error**	0.07	**Error**	0
