# Correction: Grenez, F., *et al.* Wireless Prototype Based on Pressure and Bending Sensors for Measuring Gait Quality. *Sensors* 2013, *13*, 9679–9703

**DOI:** 10.3390/s131115861

**Published:** 2013-11-21

**Authors:** Florent Grenez, María Viqueira Villarejo, Begoña García Zapirain, Amaia Méndez Zorrilla

**Affiliations:** 1 Computer Science Department, ICAM University, 6 rue Auber-59000 Lille, France; 2 DeustoTech-Life Unit, Deusto Institute of Technology, University of Deusto, Avda. de las Universidades, 24, Bilbao 48007, Spain; E-Mail: mbgarciazapi@deusto.es

In [1], we would like to change “Gate” to “Gait” in the title, which should read “Prototype Based on Pressure and Bending Sensors for Measuring Gait Quality”. In Figure 7 we would like to change the analog inputs. The measurements should be between the sensor and the resistance, and not after the resistance. The revised figure is shown below.

## Figures and Tables

**Figure 7.**
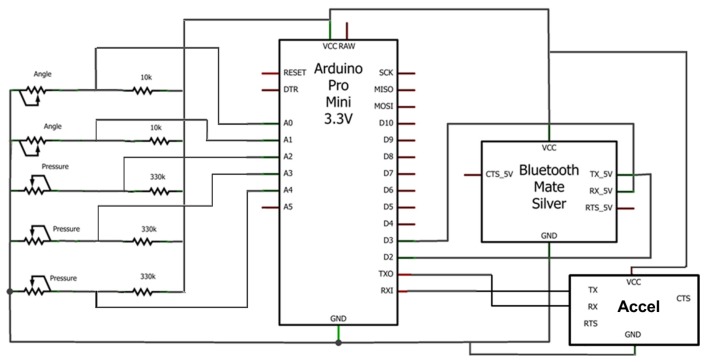
General circuit.
